# Online Racial Discrimination, Suicidal Ideation, and Traumatic Stress in a National Sample of Black Adolescents

**DOI:** 10.1001/jamapsychiatry.2023.4961

**Published:** 2024-01-03

**Authors:** Brendesha M. Tynes, Ashley Maxie-Moreman, Tuyet-Mai Ha Hoang, Henry A. Willis, Devin English

**Affiliations:** 1 Center for Empowered Learning and Development With Technology, University of Southern California, Los Angeles; 2 Division of Infectious Diseases, Children’s National Hospital, Washington, DC; 3 School of Social Work, University of Illinois at Urbana-Champaign, Urbana; 4 Department of Psychology, University of Maryland, College Park; 5 Department of Urban-Global Public Health, Rutgers University, Newark, New Jersey

## Abstract

**Importance:**

Because of increased suicide rates among Black youth in the past 2 decades, there is a dire need for research on suicidal ideation and risk factors in this population.

**Objective:**

To examine the direct and indirect associations between online racial discrimination and suicidal ideation through posttraumatic stress disorder symptoms among Black adolescents living in the US, with consideration of potential differential associations by gender and age.

**Design, Setting, and Participants:**

This cross-sectional study used data drawn from the first wave of the National Survey of Critical Digital Literacy. Black adolescents aged 11 to 19 years were selected from a nationally representative probability-based sample. Data were collected from October 2020 to December 2020 and analyzed from August 2021 to October 2021.

**Main Outcomes and Measures:**

Hypotheses of the current study were formulated during research design and grounded in empirical literature. The individual online racial discrimination subscale (Online Victimization Scale), the UCLA Child/Adolescent posttraumatic stress disorder Reaction Index for *DSM-5*, and an item from the second edition of the Children’s Depression Inventory were used to assess constructs. Mediation was assessed through mediation models with path analyses using structural equation modeling.

**Results:**

Among a total 525 participants, 265 were girls (50.5%) and 251 were boys (47.8%); the mean (SD) age was 14.8 (2.5) years. Findings from structural equation modeling analysis indicated that individual online racial discrimination was associated with posttraumatic stress disorder symptoms (β = 0.49, SE = 0.06, *P* < .001), and posttraumatic stress disorder symptoms were associated with suicidal ideation (β = 0.51, SE = 0.06, *P* < .001). Posttraumatic stress disorder was identified as a full mediator between online racial discrimination and suicide (β = 0.25, SE = 0.04, *P* < .001). No differences by gender or age were found. Furthermore, no significant direct association between online racial discrimination and suicidal ideation was found.

**Conclusions and Relevance:**

This study found an association between individual online racial discrimination and posttraumatic stress disorder symptoms and between posttraumatic stress disorder symptoms and suicidal ideation. These risk factors are important to consider in continuing studies of the cause of suicidal ideation for Black adolescents in the US.

## Key Points

**Question:**

Does individual online racial discrimination (ORD) predict suicidal ideation through posttraumatic stress disorder (PTSD) symptoms in Black adolescents based in the US, with consideration of differential associations by gender and age?

**Findings:**

In this cross-sectional study that included 525 Black adolescents, there was an indirect association between individual ORD and suicidal ideation through PTSD symptoms. This association was found for both adolescent boys and adolescent girls and across age groups.

**Meaning:**

It may be important to further examine individual ORD and PTSD symptoms to better understand the etiology of suicidal ideation for Black adolescents in the US.

## Teaser

This cross-sectional study examines data from the National Survey of Critical Digital Literacy for Black adolescents to gauge the direct and indirect associations between online racial discrimination and suicidal ideation through posttraumatic stress disorder symptoms.

## Introduction

The suicide rate among Black youth has increased over the past 2 decades.^1,2^ Although risk factors for suicidal ideation and attempts have been identified (eg, peer victimization, anxiety and depressive symptoms), meta-analyses show that the etiology of suicidal ideation and behaviors remain unclear,^3^ especially for Black youth.^2^ The theory by Opara et al^4^ conceptualizing suicide among Black youth underscores how racial minority–specific stressors such as racial discrimination may be risk factors for suicidal ideation and suicide behaviors. Extant research supports this theory, with results showing that offline racial discrimination is linked to increased suicidal ideation among Black youth.^5,6^ In addition, cyberbullying is associated with increased suicidal ideation,^7^ and Black adolescents have an average of 5 racially discriminatory experiences daily, mostly occurring online.^8^ Research is needed to determine whether online racial discrimination (ORD), defined as demeaning or excluding an individual or group on the basis of race through the use of symbols, video, images, text, and voice, may contribute to increased suicidal ideation among Black youth.

Research is also needed to better understand the mechanisms through which ORD is linked to suicidal ideation. Results from extant studies^3,9^ provide support for determining whether posttraumatic stress disorder (PTSD) is a mechanism through which experiences with ORD may contribute to suicidal ideation among Black youth and suggest potential moderation by gender.^6^ This study sought to determine whether individual ORD is associated with suicidal ideation through PTSD symptoms (hereafter referred to as PTSD) among a national sample of Black adolescents. Following Opara et al,^4^ individual ORD was expected to be associated with PTSD and suicidal ideation. We also expected PTSD to partially mediate the association between individual ORD and suicidal ideation, and given age- and gender-related disparities,^1^ stronger mediation models for girls than for boys and early and middle adolescents compared with late.

## Methods

This study was reviewed and approved by the University of Southern California institutional review board. Online informed consent and assent were obtained from participants. Data were taken from the first wave of a multimethod 2-wave longitudinal online survey, the National Survey of Critical Digital Literacy. Data were collected from October 2020 to December 2020 and analyzed from August 2021 to October 2021. Information on recruitment, measures, sampling, and weighting of data is provided in the eMethods in Supplement 1. Participants were Black adolescents categorized into 3 groups by age: early (11-13 years; 34.9%), middle (14-17 years; 49%), and late (18-19 years; 16.2%) adolescence.^10^

### Measures

Adolescent experiences with ORD were assessed using the Individual ORD subscale in the Online Victimization Scale (α = .74).^11^ Symptoms of PTSD were assessed with the UCLA Child/Adolescent PTSD Reaction Index for *DSM-5* (α = .95).^12^ Suicidal ideation was assessed with 1 item from the Children’s Depression Inventory–Short.^13^

### Statistical Analysis

Missing data (<1%) were dealt with automatically by the full information maximum likelihood method. To test our hypotheses, we conducted structural equation modeling analysis using Mplus version 8.6, which provided a framework for mediation analysis. Mediation was assessed through mediation models with path analyses using structural equation modeling. We used a robust weighted least-squares estimator, bias-corrected bootstrapping with 1000 resamples, and the multigroup function in Mplus to explore variations across gender and age.^14^ One-tailed *P* values were calculated with *P* < .05 considered significant.

## Results

Among a total 525 participants, 265 were girls (50.5%) and 251 were boys (47.8%); the mean (SD) age was 14.8 (2.5) years (Table 1). Factor loadings for 4 indicators of ORD ranged from 0.59 to 0.72 (see Table 2 for model fit). Model 1 in Table 2 was the original base mediation model and provided excellent model fit. The indirect association for the total sample (model 1) was significant (β = 0.25, SE = 0.04, *P* < .001) (Table 3). Overall, findings from the confirmatory factor analysis base mediation model (model 1) indicated that ORD was a significant predictor of PTSD (β = 0.49, SE = 0.06, *P* < .001), and PTSD was a significant predictor of suicidal ideation (β = 0.51, SE = 0.06, *P* < .001). Despite the significant bivariate correlation between ORD and suicidal ideation (Table 4), model 1 showed an indirect association between ORD on suicidal ideation through PTSD.

**Table 1.** Demographic Data for the Study Population (N = 525)

Characteristic	No. (%)^a^
Gender	
Girl	265 (50.5)
Boy	251 (47.8)
Age group, y	
11-13	183 (34.9)
14-17	257 (49)
18-19	85 (16.2)
Parent education reported by parents	
Less than high school	22 (4.2)
High school degree	117 (22.3)
High school degree and some college	135 (25.7)
Associate’s degree	93 (17.7)
Bachelor’s degree	90 (17.1)
Master’s degree or higher	68 (13)
Location of residence, US region	
Western	47 (9)
Northeastern	72 (13.7)
Midwestern	99 (18.7)
Southern	307 (58.5)
Household income	
≤$29 999	170 (32.4)
$30 000-$59 999	152 (28.9)
$60 000-$99 999	124 (23.7)
>$100 000	79 (15)

^a^ Percentages do not sum to 100 because of missing data for some characteristics.

**Table 2.** Model Comparisons

Model tested	χ^2^	*df*	Δχ^2^	CFI	ΔCFI^a^	TLI	RMSEA (90% CI)
Model 1: CFA mediation for online racial discrimination^b^	6.66	8	NA	1.00	NA	1.00	0.00 (0.00-0.05)
**Multiple group comparison models by gender**							
Model 1A: Separate by gender (girl and boy)^c^	21.28	22	NA	1.00	NA	1.00	0.00 (0.00-0.05)
Model 1B: Complete constrained model by gender^d^	22.41	25	2.76	1.00	<.001	1.00	0.00 (0.00-0.04)
**Multiple group comparison models by age**							
Model 1C: Separate by age (early, middle, and late)^e^	49.93	36	NA	0.96	NA	0.96	0.05 (0.00-0.08)
Model 1D: Complete constrained model by age^f^	71.07	42	19.37	0.93	0.03	0.93	0.06 (0.04-0.09)
Model 1F: Partial constrained model by age (ORD to PTSD and ORD to suicidal ideation now unconstrained for middle adolescents)^g^	55.21	40	5.92	0.96	0.003	0.96	0.05 (0.00-0.08)

Abbreviations: CFA, confirmatory factor analysis; CFI, comparative fit index; NA, not applicable; ORD, online racial discrimination; PTSD, symptoms of posttraumatic stress disorder; RMSEA, root mean square error of approximation; TLI, Tucker-Lewis index.

^a^ Model comparison criteria: ΔCFI and ΔTLI decreases <0.01; ΔRMSEA increases <0.015.

^b^ Model 1 is the original base CFA mediation model with the whole sample.

^c^ Model 1A estimates model 1 separately for the girls and boys (unconstrained model).

^d^ Model 1B estimates model 1 with all paths between girls and boys constrained to be equal.

^e^ Model 1C estimates model 1 separately for early, middle, and late adolescents (unconstrained model).

^f^ Model 1D estimates model 1 with all paths between early, middle, and late adolescents constrained to be equal.

^g^ Model 1F estimates model 1 only with paths constrained to be equal, excluding the middle adolescent age group, in which paths from ORD to PTSD and ORD to suicidal ideation were unconstrained as they were different from other age groups.

**Table 3.** Indirect Associations

Unique indirect association^a^	Standardized indirect association		Unstandardized indirect association		
	β	*P* value	*B* (95% CI)	SE	*P* value
In model 1 for total sample	0.25	<.001	0.25 (0.18-0.34)	0.04	<.001
In model 1B for girl and boy sample	0.24	<.001	0.24 (0.17-0.35)	0.03	<.001
In model 1F for early adolescent sample	0.19	.07	0.19 (0.12-0.28)	0.07	.006
In model 1F for middle adolescent sample	0.39	.13	0.39 (0.26-0.57)	0.13	.002
In model 1F for late adolescent sample	0.19	.07	0.19 (0.12-0.28)	0.07	.006

Abbreviations: β, standardized effect size; *B*, unstandardized effect size.

^a^ For every association, the predictor is online racial discrimination, mediator is posttraumatic stress disorder symptoms, and outcome is suicidal ideation.

**Table 4.** Pearson Correlations and Cronbach α for Variables of Interest

Variable^a^	No. of cases available	Pearson correlation				α
		Variable 1	Variable 2	Variable 3	Mean (SD)	
1. Online racial discrimination	524	1	NA	NA	0.89 (0.97)	.74
2. Symptoms of posttraumatic stress disorder	523	0.42^b^	1	NA	0.86 (0.70)	.95
3. Suicidal ideation	519	0.21^b^	0.47^b^	1	1.15 (0.39)	NA

Abbreviation: NA, not applicable.

^a^ Possible unstandardized range for online racial discrimination was 0 to 5. Possible unstandardized range for symptoms of posttraumatic stress disorder was 0 to 4. Suicidal ideation is a nominal categorical variable.

^b^ *P* < .01.

Further findings in Table 2 demonstrated that the base mediation models (models 1A and 1B) were a good fit for both genders. The χ^2^ difference for model 1A and 1B was not significant (Δχ^2^ = 1.70, *P* = .64), and thus model 1B was selected as most parsimonious with good fit. Model 1B (Table 3) showed a significant indirect association for girls and boys (*B* = 0.24, SE = 0.03, *P* < .001). Findings from path regressions (ie, ORD to PTSD, ORD to suicidal ideation, and PTSD to suicidal ideation) from model 1B by gender are shown in eFigure 1 in Supplement 1. For girls, paths from ORD to PTSD (β = 0.48, SE = 0.04, *P* < .001) and from PTSD to suicidal ideation (β = 0.51, SE = 0.06, *P* < .001) were all significant. Similarly for boys, paths from ORD to PTSD (β = 0.47, SE = 0.04, *P* < .001) and from PTSD to suicidal ideation (β = 0.52, SE = 0.06, *P* < .001) were significant.

For age groups, model 1C (the unconstrained model) provided acceptable fit. Model 1D did not pass criteria for constrained paths. Model 1F passed criteria for a partial constrained model. Model 1F was selected with findings for indirect associations (Table 3) and direct association paths (eFigure 2 in Supplement 1). In model 1F, all regression paths were constrained to be equal for all age groups except for 2 paths (ORD to PTSD and ORD to suicidal ideation), which were unconstrained for middle adolescents. Findings suggested that indirect associations were significant for early (*B* = 0.19, SE = 0.07, *P* = .006), middle (*B* = 0.39, SE = 0.13, *P* = .002), and late (*B* = 0.19, SE = 0.07, *P* = .06) adolescents.

ORD significantly predicted PTSD for early (β = 0.33, SE = 0.07, *P* < .001), middle (β = 0.74, SE = 0.10, *P* < .001), and late (β = 0.34, SE = 0.08, *P* < .001) adolescents. Additionally, PTSD significantly predicted suicidal ideation for early (β = 0.57, SE = 0.12, *P* < .001), middle (β = 0.52, SE = 0.12, *P* < .001), and late (β = 0.55, SE = 0.01, *P* < .001) adolescents. ORD did not predict suicidal ideation for any age group.

## Discussion

Our hypotheses were partially confirmed in that we found direct and indirect positive associations between ORD and PTSD, PTSD and suicidal ideation, and ORD and suicidal ideation through PTSD, respectively. Contrary to our expectations informed by literature about offline discrimination that shows links between racial discrimination and suicidal ideation,^6^ we did not find a direct association between ORD and suicidal ideation. Additionally, in contrast to the offline racial discrimination literature that shows differences by gender,^4^ no variations by age or gender were found. Overall, these findings contribute to our understanding of the discrimination–suicidal ideation relationship in online settings.

### Limitations

Study limitations include the cross-sectional design, which limits our ability to make causal claims about the influence of PTSD on the discrimination–suicidal ideation link. Further, given that we use a single-item measurement of suicidal ideation, this could result in misclassification and increase in the likelihood of statistical decision errors.^15^

## Conclusions

This study found an association between individual ORD and PTSD and between PTSD and suicidal ideation. Given the increasing suicide rates among Black youth,^1,2^ online platforms should be aware of the links between ORD, PTSD, and suicidal ideation and create safer spaces for Black adolescents by proactively monitoring and reducing hate speech. Additionally, future research is needed to better understand the temporal nature of the relation among ORD, PTSD, and suicidal ideation.

## Notes

Supplement 1.
eMethods

eReferences
**eFigure 1.** Partial mediation path model for the effects of online racial discrimination and symptoms of post-traumatic stress disorder on suicide ideation among girls and boys **eFigure 2.** Partial mediation path model for the effects of online racial discrimination and symptoms of post-traumatic stress disorder on suicide ideation among three age groups

Supplement 2. Data sharing statement
