# Supplementary material for: Online Racial Discrimination, Suicidal Ideation, and Traumatic Stress in a National Sample of Black Adolescents
Source: JAMA Psychiatry. 2024 Jan 3;81(3):312–6. doi: 10.1001/jamapsychiatry.2023.4961 (PMC10765309; doi:10.1001/jamapsychiatry.2023.4961)
Supplement: Supplement 1. — eMethods eReferences eFigure 1. Partial mediation path model for the effects of online racial discrimination and symptoms of post-traumatic stress disorder on suicide ideation among girls and boys eFigure 2. Partial mediation path model for the effects of online racial discrimination and symptoms of post-traumatic stress disorder on suicide ideation among three age groups [file jamapsychiatry-e234961-s001.pdf]

Supplemental Online Content

Tynes BM, Maxie-Moreman A, Hoang TMH, Willis HA, English D. Online racial discrimination, suicidal ideation, and traumatic stress in a national sample of Black adolescents. *JAMA Psychiatry*. Published online January 3, 2024. doi:10.1001/jamapsychiatry.2023.4961

eMethods
eReferences
eFigure 1. Partial mediation path model for the effects of online racial discrimination and symptoms of post-traumatic stress disorder on suicide ideation among girls and boys
eFigure 2. Partial mediation path model for the effects of online racial discrimination and symptoms of post-traumatic stress disorder on suicide ideation among three age groups

This supplemental material has been provided by the authors to give readers additional information about their work.

© 2024 Tynes BM et al. *JAMA Psychiatry.*

eMethods. Detailed information on methods. This includes information on recruitment and selection, survey sampling methods, response rates, sample weighting, design, setting, participants, and measures.

Data in the current study were taken from the first wave of the National Survey of Critical Digital Literacy (NSCDL). Surveys were conducted on KnowledgePanel® in Ipsos. KnowledgePanel is the first and largest online research panel that is representative of the entire U.S. population. Panel members are randomly recruited through probability-based sampling, and households are provided with access to the Internet and hardware if needed.

Recruitment

Ipsos recruits panel members using address-based sampling (ABS) methods to ensure full coverage of all households in the nation. Adults from sampled households are invited to join KnowledgePanel through a series of mailings, including an initial invitation letter, a reminder postcard, and a subsequent follow-up letter. Moreover, telephone refusal-conversion calls are made to nonresponding households for which a telephone number could be matched to a physical address. Invited households can join the panel by:

- Completing and mailing back a paper form in a postage-paid envelope
- Calling a toll-free hotline phone number maintained by Ipsos
- Going to a designated Ipsos website and completing the recruitment form online

After initially accepting the invitation to join the panel, participants are asked to complete a short demographic survey (the initial Core Profile Survey); answers to this survey allow efficient panel sampling and weighting for future surveys. Upon completing the Core Profile Survey, participants become active panel members. All panel members are provided privacy and confidentiality protections.

Household Member Recruitment. During the initial recruitment survey, all household members are enumerated. Following enumeration, attempts are made to recruit every household member who is at least 13 years old to participate in KnowledgePanel surveys. For household members aged 13 to 17, consent is collected from the parents or the legal guardian during the initial recruitment interview. No direct communication with teenagers is attempted before obtaining parental consent. Once household members are recruited for the panel and assigned to a study sample, they are notified by email for survey taking, or panelists can visit their online member page for survey taking (instead of being contacted by telephone or postal mail). This allows surveys to be fielded quickly and economically. In addition, this approach reduces the burden placed on respondents, since email notification is less intrusive than telephone calls and the self- administered mode minimizes social desirability bias and positivity effects that can be present with an interviewer. Many respondents find answering online questionnaires more interesting and engaging than being questioned by a telephone interviewer. Furthermore, respondents have the convenience to choose what day and time to complete their assigned survey.

Random Selection

Panel members are randomly selected so that survey results can properly represent the U.S. population with a measurable level of accuracy and a calculable response rate, features that are not obtainable from nonprobability or opt-in online panels (for comparisons of results from probability versus nonprobability methods, see MacInnis et al., 2018[16] and Yeager et al., 2011[17]).

Survey

Survey Sampling. Once panel members are recruited and profiled by completing our Core Profile Survey, they become eligible for selection for client surveys. Typically, specific survey samples are based on an equal probability selection method (EPSEM) for general population surveys. Customized stratified random sampling based on “profile” data can also be implemented as required by the study design. Profile data can also be used when a survey calls for pre-screening—that is, members are drawn from a subsample of the panel, such as females, Republicans, grocery shoppers, etc. (This can reduce screening costs, particularly for lower incidence subgroups.) In such cases, we ensure that all subsequent survey samples drawn that week are selected in such a way as to result in a sample that remains representative of the population distributions.

Survey Administration. Once assigned to a survey, members receive a notification email letting them know there is a new survey available for them to complete. This email notification contains a link that sends them to the survey. No login name or password is required. The field period depends on the client’s needs and can range anywhere from a few hours to several weeks. Typically, after three days, automatic email reminders are sent to all non-responding panel members in the sample. Additional email reminders are sent, or custom reminder schedules are set up as needed. To assist panel members with their survey taking, everyone has a personalized member portal listing all assigned surveys that have yet to be completed.

© 2024 Tynes BM et al. *JAMA Psychiatry.*

Response Rates

As a member of the American Association of Public Opinion Research (AAPOR), Ipsos follows the AAPOR standards for response rate reporting. While the AAPOR standards were established for single survey administrations and not for multi-stage panel surveys, we use the Callegaro-DiSogra (2008)[18] algorithms for calculating KnowledgePanel survey response rates.

Sample Weighting

As detailed above, significant resources and infrastructure are devoted to the recruitment process for KnowledgePanel so that our active panel members can properly represent the population of the U.S. This representation is achieved not only with respect to a broad set of geodemographic indicators, but also for hard-to-reach (such as those without Internet access or Spanish- language-dominant Hispanics) who are recruited in proper proportions. Consequently, the raw distribution of KnowledgePanel mirrors that of the U.S. adults fairly closely, barring occasional disparities that emerge for certain subgroups due to differential recruitment and attrition.

For selection of general population samples from KnowledgePanel, a patented methodology has been developed that ensures all samples behave as EPSEM samples. Briefly, this methodology starts by weighting the pool of active members to the geodemographic benchmarks secured from a combination of the U.S. Census Bureau's American Community Survey (ACS) and the latest March supplement of the U.S. Census Bureau's Current Population Survey (CPS) along several dimensions. Using the resulting weights as measures of size, a probability-proportional-to-size (PPS) procedure is used to select study specific samples. It is the application of this PPS methodology with the imposed size measures that produces demographically balanced and representative samples that behave as EPSEM. Moreover, in instances where a study design requires any form of oversampling of certain subgroups, such departures from an EPSEM design are accounted for by adjusting the design weights in reference to the Census benchmarks for the population of interest. Typically, the geodemographic dimensions used for weighting the entire KnowledgePanel include the following dimensions, with additional nesting of dimensions as well:

- Gender (Male/Female)
- Age (18–29, 30–44, 45–59, and 60+)
- Race/Hispanic ethnicity (White/Non-Hispanic, Black/Non-Hispanic, Other/Non-Hispanic, 2+ Races/Non-Hispanic, Hispanic)
- Education (Less than High School, High School, Some College, Bachelor and beyond)
- Census Region (Northeast, Midwest, South, West)
- Household income (under $10k, $10K to <$25k, $25K to <$50k, $50K to <$75k, $75K to
- <$100k, $100K to <$150k, and $150K+)
- Home ownership status (Own, Rent/Other)
- Household size (1, 2, 3, 4+)
- Metropolitan Area (Yes, No)
- Hispanic Origin (Mexican, Puerto Rican, Cuban, Other, Non-Hispanic)
- Language Dominance (non-Hispanic and English Dominant, Bilingual, and Spanish Dominant Hispanic) when survey is administered in both English and Spanish

NSCDL Study-Specific Post-Stratification Weights. Once all survey data have been collected and processed, design weights are adjusted to account for any differential nonresponse that may have occurred. Depending on the specific target population for a given study, geodemographic distributions for the corresponding population are obtained from the CPS, the U.S. Census Bureau's American Community Survey (ACS), or in certain instances from the weighted KnowledgePanel profile data. For this purpose an iterative proportional fitting (raking) procedure is used to produce the final weights. In the final step, calculated weights are examined to identify and, if necessary, trim outliers at the extreme upper and lower tails of the weight distribution. The resulting weights are then scaled to aggregate to the total sample size of all eligible respondents. For this study, our weighting process included the following steps:

1. In the first step, design weights for all KnowledgePanel (KP) parent assignees were computed to reflect their selection probabilities.

© 2024 Tynes BM et al. *JAMA Psychiatry.*

2. We then multiplied the parents' design weights by the number of 11-19 year old teens in the household (1 vs 2+ based on QS2) to account for the probability of teen selection. These served as the design weights for KP teen respondents. These design weights for KP teen respondents were raked to the following geodemographic distributions of the age 11-19 White/Non-Hispanic, African- American/Non-Hispanic (including biracial African-Americans), and Hispanic population with finer geo-demographic adjustments within the three race-ethnicity groups. The benchmarks were obtained from the 2019 American Community Survey (ACS). The metropolitan status benchmarks were obtained from the 2020 March Supplement of the Current Population Survey (CPS).

- Gender (Male, Female) by Age (11-13, 14-16, 17-19)
- Race/Hispanic ethnicity (White/Non-Hispanic, African-American/Non-Hispanic (including biracial African-Americans), Hispanic)
- Census Region (Northeast, Midwest, South, West)
- Metropolitan status (Metro, Non-Metro)
- Household income (Under $25K, $25K-$49,999, $50K-$74,999, $75K-$99,999, $100K – $149,999, $150K+)

We started with a weight of 1.0 for qualified opt-in respondents and multiplied the weights by the number of 11-19 year old teens in the household to account for the probability of teen selection. Teens were then weighted to resemble the benchmarks for parents of 11-19 year old non-Hispanic African-Americans (include biracial African-Americans). The benchmarks of the calibration variables are based on the weighted KP respondents.

- Gender (Male, Female) by Age (11-13, 14-16, 17-19)
- Census Region (Northeast, Midwest, South, West) c Metropolitan status (Metro, Non-Metro)
- Household income (Under $25K, $25K-$49,999, $50K-$74,999, $75K-$99,999, $100K +)
- QCP0003 - Watch TV (< 4 hours/day, 4+ hours/day)
- QCP0005 - Internet for Personal Use (< 6 hours/week, 6+ hours/week)

KP and opt-in teen respondents were first combined based on their respective effective sample sizes. Then, the combined teen respondents were weighted to represent the age 11-19 White/Non- Hispanic, African-American/Non-Hispanic (including biracial African-Americans) and Hispanic population on the same variables as in step 2 above and calibration variables within African- American. The resulting weights were trimmed and scaled to sum to the unweighted sample size of total respondents (labeled as weight).

- Gender (Male, Female) by Age (11-13, 14-16, 17-19)
- Race/Hispanic ethnicity (White/Non-Hispanic, African-American/Non-Hispanic (including biracial African-Americans), Hispanic)
- Census Region (Northeast, Midwest, South, West)
- Metropolitan status (Metro, Non-Metro)
- Household income (Under $25K, $25K-$49,999, $50K-$74,999, $75K-$99,999, $100K - $149,999, $150K+)
- QCP0003 - Watch TV (< 4 hours/day AA, 4+ hours/day by White/Non-Hispanic , African- American/Non-Hispanic, Hispanic)
- QCP0005 - Internet for Personal Use (< 6 hours/week AA, 6+ hours/week by White/Non-Hispanic, African-American/Non-Hispanic, Hispanic)

Weights Definition

- kp_weight: Weights for KP respondents   (interim weights not delivered)
- offpanel_weight: Weights for Opt-In respondents (interim weights not delivered)
- weight: Weights for total KP and Opt-In respondents
- weight_White: Weights for total KP and Opt-In White respondents

© 2024 Tynes BM et al. *JAMA Psychiatry.*

- weight_Black: Weights for total KP and Opt-In AA respondents
- weight_Hispnic: Weights for total KP and Opt-In Hispanic respondents

Trimming:

- kp_weight: None
- offpanel_weight: (0%, 99.22%)
- weight: None
- weight_White: scaled from weight
- weight_Black: scaled from weight
- weight_Hisp: scaled from weight

Design Effect

- kp_weight: 1.3788
- offpanel_weight: 1.6795
- weight: 1.5980
- weight_White: 1.1679
- weight_Black: 1.4783
- weight_Hisp: 1.1861

Design, Participants, and Measures

Design and Setting. Data were taken from the first wave of a multi-method two-wave longitudinal online survey, the National Survey of Critical Digital Literacy (NSCDL). Surveys were conducted on KnowledgePanel, the largest panel online in the U.S., that used a probability-based sampling method for recruitment. The nationally representative sample was recruited only in the U.S. (N = 1,138) and data were collected in 2020.

Participants. The current study included 525 participants ages 11 to 19 who identified as Black, and participants were equally divided between identified cis-gendered girls (50.5%) and boys (47.8%) (see Table 1 for descriptive statistics). Participants were categorized into three groups: early (11-13 years; 34.9%), middle (14-17 years; 49%), and late adolescent (18-19 years; 16.2%).[10]

Measures

Online Racial Discrimination. Adolescents' experiences with ORD were assessed using the Individual ORD subscale in the Online Victimization Scale.[11] This subscale measures discriminatory text, images, or symbols that directly target an individual for their race. The Individual ORD subscale includes four original items (e.g., "People have said mean or rude things about me because of my race or ethnic group online"). Responses assess exposure to ORD over a 12-month period and range from 0 = *Never* to 5 = *Every day*. In this study, the scale exhibited good reliability (α=.74).

Post-traumatic Stress Disorder Symptoms. PTSD symptoms were assessed with the UCLA Child/Adolescent PTSD Reaction Index for *DSM-5* (UCLA PTSD-RI-5).[12] This measure assesses post-traumatic stress symptoms in children and adolescents (e.g., "I have trouble concentrating or paying attention."). Responses range from 0 = *None of the time* to 4 = *Most of the time*. In this study, the measure showed good reliability (α=.95).

Suicide ideation. SI was assessed with one item from the Children's Depression Inventory – Short (CDI – S).[13] Respondents were asked to endorse one of the following options: "I do not think about killing myself" (coded as 1), "I think about killing myself, but wouldn't do it" (coded as 2), and "I want to kill myself" (coded as 3).

© 2024 Tynes BM et al. *JAMA Psychiatry.*

eReferences

1. Sheftall AH, Vakil F, Ruch DA, Boyd RC, Lindsey MA, Bridge JA. Black youth suicide: Investigation of current trends and precipitating circumstances. *Journal of the American Academy of Child & Adolescent Psychiatry.* 2021. doi: 10.1016/j.jaac.2021.08.021

2. *Ring the Alarm: The Crisis of Black youth Suicide in America.* Congressional Black Caucus. 2020:1-41. A Report to Congress from the Congressional Black Caucus, Emergency Taskforce on Black Youth Suicide and Mental Health. Accessed October 5, 2021. https://theactionalliance.org/sites/default/files/ring_the_alarm-_the_crisis_of_black_youth_suicide_in_america_copy.pdf

3. Bentley KH, Franklin JC, Ribeiro JD, Kleinman EM, Fox KR, Nock MK. Anxiety and its disorders as risk factors for suicidal thoughts and behaviors: A meta-analytic review. *Clinical Psychology Review.* 2016; 30-46. doi: 10.106/j.cpr.2015.11.008

4. Opara I, Assan MA, Pierre K, Gunn III JF, Metzger I, Hamilton J, Arugu E. Suicide among Black children: An integrated model of the interpersonal-psychological theory of suicide and intersectionality theory for researchers and clinicians. *Journal of Black Studies.* 2020; 51(6): 611-631. doi: 10.1177/0021934720935641

5. Assari S, Lankarani MM, Caldwell CH. Discrimination increases suicide ideation in Black adolescents regardless of ethnicity and gender. *Behavioral Sciences.* 2017;7(4):75. doi: 10.3390/bs7040075

6. Walker R, Francis D, Brody G, Simons R, Cutrona C, Gibbons F. A longitudinal study of racial discrimination and risk for death ideation in African American youth. *Suicide and Life-Threatening Behavior.* 2017;47(1): 86-102. doi: 10.1111/sltb.12251

7. John A, Glendenning AC, Marchant A., et al. Self-harm, suicidal behaviours, and cyberbullying in children and young people: Systematic review. *Journal of Medical Internet Research.* 2018;20(4). Accessed October 5, 2021. https://preprints.jmir.org/preprint/9044

8. English D, Lambert SF, Tynes BM, Bowleg L, Zea MC, Howard LC. Daily multidimensional racial discrimination among Black U.S. American adolescents. Journal of Applied Developmental Psychology. 2020; 66: 101068. doi: 10.1016/j.appdev.2019.101068

9. Maxie-Moreman A, Tynes BM. Exposure to online racial discrimination and traumatic events online in Black adolescents and emerging adults. *Journal of Research on Adolescence*. 2022;31(1):254-269. doi: 10.1111/jora.12732

10. Salmela-Aro K. Stages of adolescence. *Encyclopedia of Adolescence.* 2011;360-368. doi: 10.1016/B978-0-12-

373951-3.00043-0

11. Tynes BM, Rose CA, Williams DR. The development and validation of the online victimization scale for adolescents. *Journal of Psychosocial Research on Cyberspace.* 2010;4(2). Accessed October 5, 2021. https://cyberpsychology.eu/article/view/4237

12. Roulon-Arroyo B, Oosterhoff B, Layne CM, Steinberg AM, Pynoos RS, Kaplow JB. The UCLA PTSD reaction index for DSM-5 brief form: A screening tool for trauma-exposed youths. *Journal of the American Academy of Child and Adolescent Psychiatry.* 2019;59(3):434-443. doi: 10.1016/j.jaac.2019.06.015

13. Kovacs M. Children's depression inventory (CDI and CDI 2). *The Encyclopedia of Clinical Psychology.* 2015. doi: 10.1002/9781118625392.wbecp419

14. Napolitano CM, Job V. Assessing the implicit theory of willpower for strenuous mental activities scale: Multigroup, across-gender, and cross-cultural measurement invariance and convergent and divergent validity. *Psychological Assessment.* 2018;30(8). doi: 10.1037/pas0000557

15. Millner AJ, Lee MD, Nock MK. Single-item measurement of suicidal behaviors: Validity and consequences of misclassification. *PLOS ONE*. 2015;10(10): e0141606. doi: 10.1371/journal.pone.0141606

16. MacInnis B, Krosnick J, Ho A, Cho M. The accuracy of measurements with probability and nonprobability survey samples: Replication and extension. *Public Opinion Quarterly.* 2018;82(4):707-744. doi: 10.1093/poq/nfy038

17. Yeager, D, Krosnick, J, Chang, L, Javitz, H, Levendusky, M, Simper, A, Wang, R. Comparing the accuracy of RDD telephone surveys and internet surveys conducted with probability and non-probability samples. *Public Opinion Quarterly.* 2011;75(4):709-747. doi: 10.1093/poq/nfr020

18. Callegaro M, DiSogra C. Computing response metrics for online panels. *Public Opinion Quarterly*. 2008;72(5): doi: 10.1093/poq/nfn065

© 2024 Tynes BM et al. *JAMA Psychiatry.*

eFigure 1. Partial mediation path model for the effects of online racial discrimination and symptoms of post-traumatic stress disorder on suicide ideation among girls and boys. All path effects reflect standardized estimates. *$p \leq .05$; **$p < .01$.

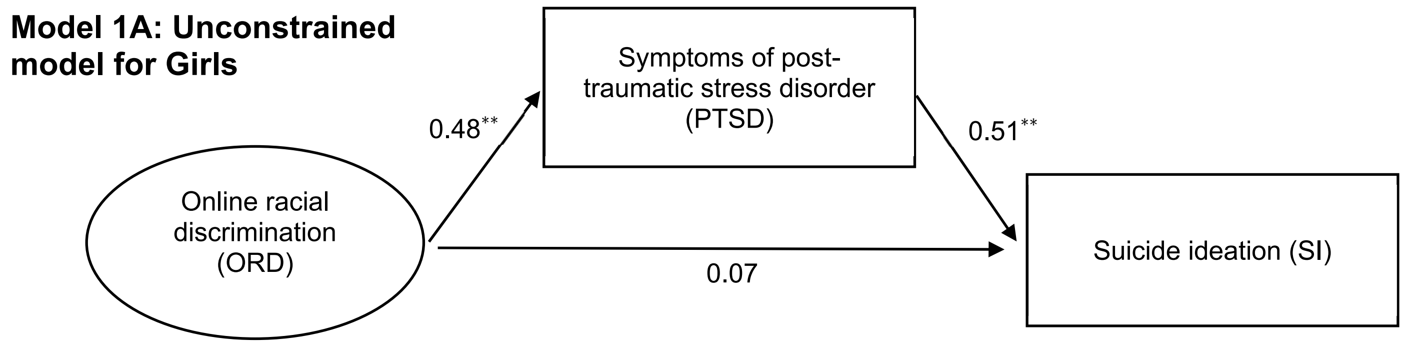

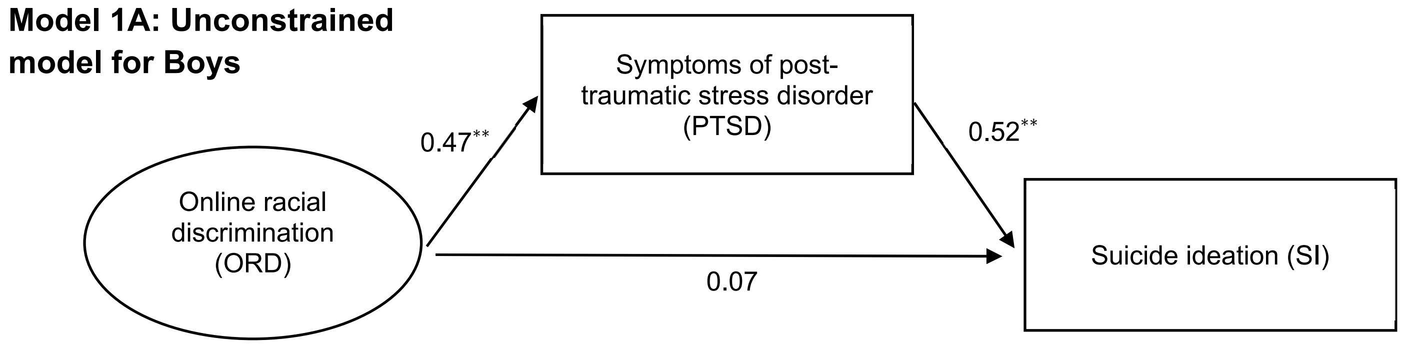

© 2024 Tynes BM et al. *JAMA Psychiatry.*

eFigure 2. Partial mediation path model for the effects of online racial discrimination and symptoms of post-traumatic stress disorder on suicide ideation among three age groups. All path effects reflect standardized estimates. *$p \leq .05$; **$p < .01$.

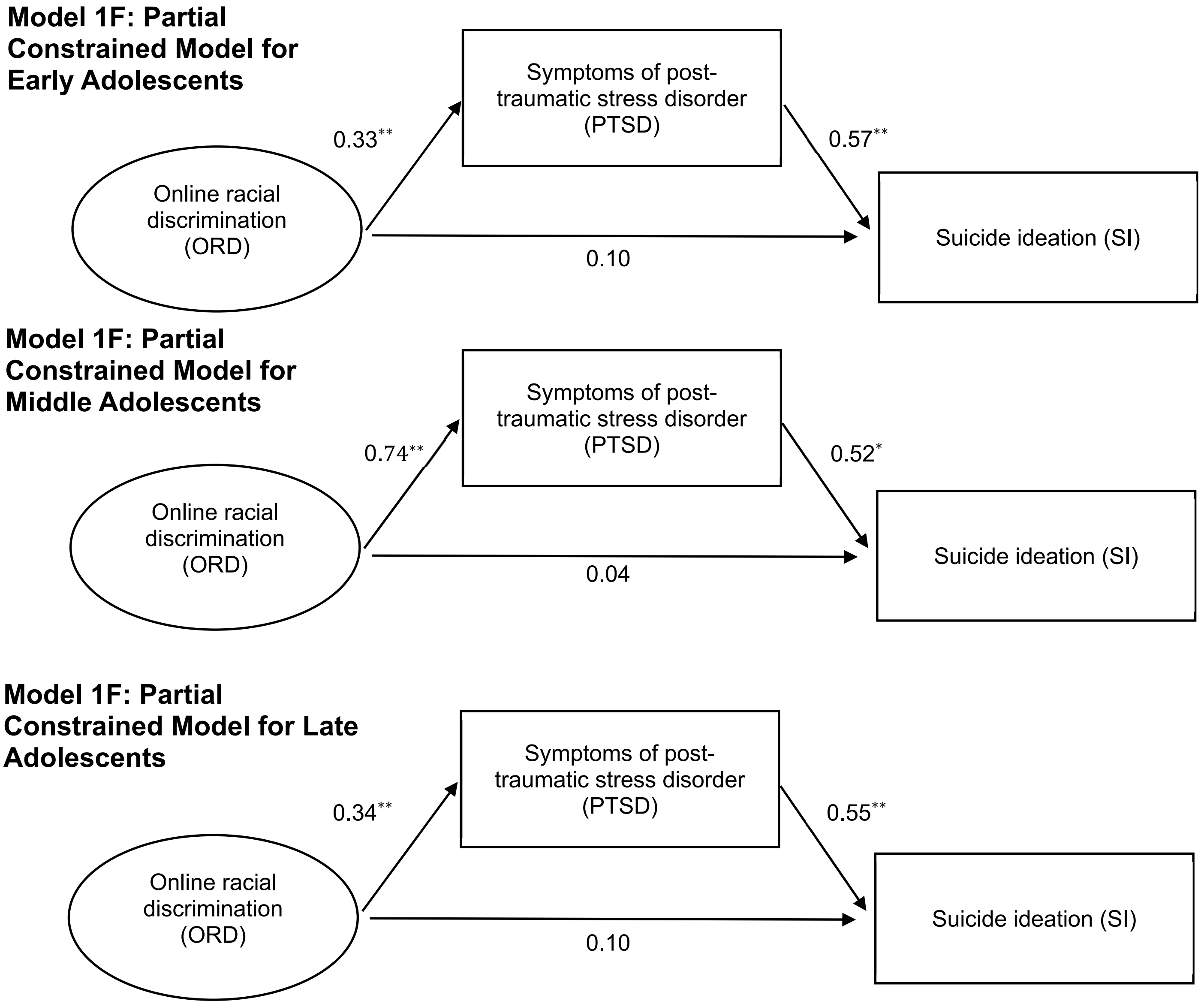

© 2024 Tynes BM et al. *JAMA Psychiatry.*
