# Supplementary material for: Online Racial Discrimination, Suicidal Ideation, and Traumatic Stress in a National Sample of Black Adolescents
Source: JAMA Psychiatry. 2024 Jan 3;81(3):312–6. doi: 10.1001/jamapsychiatry.2023.4961 (PMC10765309; doi:10.1001/jamapsychiatry.2023.4961)
Supplement: Supplement 2. — Data sharing statement [file jamapsychiatry-e234961-s002.pdf]

Data Sharing Statement

Tynes. Online Racial Discrimination, Suicidal Ideation, and Traumatic Stress in a National Sample of Black Adolescents. *JAMA Psychiatry*. Published January 03, 2024. doi:10.1001/jamapsychiatry.2023.4961

Data
Data available: No
